# Transcriptome Sequencing Highlights the Regulatory Role of DNA Methylation in Immune-Related Genes’ Expression of Chinese Oak Silkworm, *Antheraea pernyi*

**DOI:** 10.3390/insects13030296

**Published:** 2022-03-17

**Authors:** Saima Kausar, Ruochen Liu, Isma Gul, Muhammad Nadeem Abbas, Hongjuan Cui

**Affiliations:** 1 State Key Laboratory of Silkworm Genome Biology, Key Laboratory of Sericultural Biology and Genetic Breeding, Ministry of Agriculture, Southwest University, Chongqing 400716, China; drkausarsn@hotmail.com (S.K.); biolrc@126.com (R.L.); ismagul@163.com (I.G.); 2 Cancer Center, Medical Research Institute, Southwest University, Chongqing 400716, China

**Keywords:** Chinese oak silkworm, DNA methylation, microbial infection, innate immunity

## Abstract

**Simple Summary:**

*Antheraea pernyi* is an important lepidopteran that has been used as a model insect for various physiological systems. DNA methylation has recently been discovered to control a variety of physiological processes in animals throughout their lives. We investigated the effect of DNA methylation on the innate immune system of *A. pernyi* and found that DNA methylation inhibition modifies gene expression, especially in immune-related genes. Finally, our data suggest that DNA methylation may regulate gene expression in insects, and microbial infection likely induces DNA methylation of the host genome.

**Abstract:**

*Antheraea pernyi* is an important lepidopteran used as a model insect species to investigate immune responses, development, and metabolism modulation. DNA methylation has recently been found to control various physiological processes throughout the life of animals; however, DNA methylation and its effect on the physiology of insects have been poorly investigated so far. In the present study, to better understand DNA methylation and its biological role in the immune system, we analyzed transcriptome profiles of *A. pernyi* pupae following DNA methylation inhibitor injection and Gram-positive bacteria stimulation. We then compared the profiles with a control group. We identified a total of 55,131 unigenes from the RNA sequence data. A comparison of unigene expression profiles showed that a total of 680 were up-regulated and 631 unigenes were down-regulated in the DNA-methylation-inhibition-bacteria-infected group compared to the control group (only bacteria-injected pupae), respectively. Here, we focused on the immune-related differentially expressed genes (DEGs) and screened 10 genes that contribute to immune responses with an up-regulation trend, suggesting that microbial pathogens evade host immunity by increasing DNA methylation of the host genome. Furthermore, several other unigenes related to other pathways were also changed, as shown in the KEGG analysis. Taken together, our data revealed that DNA methylation seems to play a crucial biological role in the regulation of gene expression in insects, and that infection may enhance the host genome DNA methylation by a yet-unknown mechanism.

## 1. Introduction

DNA methylation is an epigenetic molecular mechanism that modulates various physiological processes in mammals and plants, such as gene regulation, DNA repair, development, tumorigenesis, and nutrigenomics [1,2,3]. Recent studies have also shown that DNA methylation has a crucial biological role in host immune responses. For example, viruses, especially DNA tumor viruses, can stimulate aberrant DNA methylation of the host genome to reduce the host immune responses and thereby evade antiviral immunity [4,5,6]. Viruses also affect host DNA methyltransferase expression patterns, the DNA methylation regulators for epigenetic dysregulation of immune-associated gene expression in host cells [7,8]. Interestingly, it has been shown that demethylation treatment of cancer cells can activate the viral RNA transcription from dormant endogenous retroviruses and trigger antiviral signaling [9,10]. In comparison to mammals, DNA methylation and its effect on different physiological activities of insects have been poorly investigated. Recent progress in DNA methylation research methods has prompted researchers to analyze the role of DNA methylation in insects. Whole-genome bisulfite sequencing studies on different insect species, e.g., *Bombyx mori, Tribolium castaneum, Drosophila melanogaster*, and *Nasonia vitripennis* [11,12,13,14], show that DNA methylation exists in the insect genome [15,16,17]. DNA methylation has been linked to immunity, aging, evolution, memory, and caste determination modulation in bees and other insect species [18,19,20,21,22,23,24]. 

The biological role of DNA methylation in innate immunity in insects has not been explored well. In the *Aedes aegypti*, genome-wide patterns of DNA methylation were disrupted after Wolbachia infection [25]. Similarly, *Metarhizium anisopliae* infection caused the differential expression of DNA methyltransferase (DNMT) genes in the larvae of *Galleria mellonella* [17]. *Bombyx mori* cytoplasmic polyhedrosis virus (BmCPV) infection in *Bombyx mori* induced the 27 gene differential expression and differential methylation in the midgut and fat body of infected larvae, respectively, indicating that BmCPV infection modifies the gene expression by mediating variations in DNA methylation [26]. These results suggest that DNA methylation may play a crucial biological role in the immune response of insects against pathogens. However, besides the importance of DNA methylation, this mechanism has poorly been studied, underscoring the indispensability of DNA methylation in insect physiology. 

The Chinese oak silkworm, *A. pernyi,* has been used as a model insect species to study immune response, development, breeding technique, and metabolism regulation. In addition, it is cultured in various Asian countries, including China, Korea, and India, for silk production and as a highly nutritious food [27,28]. Thus, investigating different molecular mechanisms is important to understand the physiological responses of this species and that of other insects. In the present study, we construct a transcriptome-sequencing library from the fat bodies of *A. pernyi* after administrating DNA methylation inhibitors and a bacterial challenge and then compare it with the control group. The results are interesting, indicating the presence of DNA methylation in *A. pernyi* that regulates gene differential expression, especially of immune-related genes.

## 2. Materials and Methods

### 2.1. Experimental Insects 

The Sericulture Research Institute in Henan, China, provided *A. pernyi* larvae, which were then fed fresh oak leaves and kept at the State Key Laboratory of Silkworm Genome Biology, Southwest University, Chongqing, China. They were kept at room temperature (21–26 °C), with a light/dark photoperiod of 10/14 h and 70% relative humidity. These larvae were allowed to transform into pupae, which were maintained at room temperature. 

### 2.2. 5-Azacytidine Administration, Bacterial Challenge, and Tissue Collection

We divided the pupae into two groups of nine. 5-azacytidine (5-AZA), a DNA methylation inhibitor, and Gram-positive bacteria (*Bacillus cereus*) were injected into first group, while only Gram-positive bacteria were injected into the second. The inhibition of DNMTs was also investigated by using qRT-PCR. Although a direct effect of 5-AZA on the bacteria used in this study cannot be ruled out, previous studies showed that 5-AZA has no impact on bacterial growth at the concentration used in the present study [23]. For a bacterial challenge, we injected the bacterial cells (4 × 10^3^ cells/larva detected by the method of the colony-forming unit) and 1 μL 5-AZA (40 mM) into pupae after the fourth day since the larvae transformed to pupae. Injections were performed between the second and third tergite of the abdomen of the pupae. Bacterial infection was completed 24 h post second 5-AZA administration. The fat bodies of *A. pernyi* pupae were collected after 12 h of bacterial infection and then immediately stored in a refrigerator at −80 °C. Three pupal fat bodies were mixed together and taken as one sample, which was then repeated three times.

### 2.3. RNA Preparation, Library Construction, and Sequencing 

Total RNA was extracted from pupal fat bodies using Trizol Reagent (Invitrogen, Carlsbad, CA, USA) according to the manufacturer’s instructions. The purity of the RNA was analyzed using a Nano spectrophotometer (Implen, Westlake Village, CA, USA), and the concentration was measured using the Qubit RNA Assay Kit in a Qubit 2.0 Fluorometer (Life Technologies, Carlsbad, CA, USA). A total of six RNA libraries were constructed using Illumina TruSeq RNA preparation kits, which were assembled in accordance with the instructions provided by the manufacturer. Further verification and quantification were carried out using the Qubit dsDNA BR Assay Kit (Q32850, Invitrogen™, Carlsbad, CA, USA) and the Agilent Bioanalyzer 2100 (Agilent Technologies, Santa Clara, CA, USA). To obtain raw sequencing data in this study, the HiSeq™ 2000 Sequencing System (Illumina, San Diego, CA, USA) was employed. 

### 2.4. Transcriptome Assembly, Annotation, and Function Enrichment

The obtained raw sequencing data were processed to generate clean reads by removing reads containing adapters, poly-N, and low-quality reads using Cutadapt and Perl scripts in-house. The sequence quality, such as Q20, Q30, and GC-content of the clean reads, was confirmed by FastQC. All downstream analyses were based on clean data with high quality. Trinity software was used for de novo assembly of transcriptome data [28], and finally, unigene was obtained. The Salmon method [29] was utilized to execute the expression level for unigenes by calculating TPM [30]. The differentially expressed unigenes were selected with log2 (fold change) > 1 or log2 (fold change) < −1 and with statistical significance (*p*-value < 0.05) by the R package edger.

### 2.5. Unigene Annotation and Functional Classification

For annotation and functional classification, assembled unigenes were aligned against the non-redundant (Nr) protein database (http://www.ncbi.nlm.nih.gov/, accessed on 14 May 2021), Gene ontology (GO) (http://www.geneontology.org, accessed on 14 May 2021), Clusters of Orthologous Groups of proteins (KOG/COG), SwissProt (http://www.expasy.ch/sprot/, accessed on 14 May 2021), Kyoto Encyclopedia of Genes and Genomes (KEGG) (http://www.genome.jp/kegg/, accessed on 14 May 2021), and eggNOG (http://eggnogdb.embl.de/, accessed on 14 May 2021) databases using DIAMOND with a threshold of E-value < 0.00001. GO and KEGG enrichment analysis was again performed on the differentially expressed unigenes by Perl scripts in-house.

### 2.6. Quantitative RT-PCR Analysis 

Ten up-regulated DEGs were selected for quantitative RT-PCR to validate the transcriptome data. Total RNA was isolated from the 5-AZA+bacteria-injected samples and the control samples using Trizol solution according to the manufacturer’s instructions for cDNA synthesis, which was then used as a template for the qRT-PCR assay for the validation of RNA-Seq data. To eliminate any DNA contamination, all of the samples were treated with DNase I (Promega). The RNA concentrations were determined using a spectrophotometer, and a total of 2 µg of RNA was reverse-transcribed with Hifair^®^ III 1st Strand cDNA Synthesis SuperMix for qPCR kit. The gene-specific primers were designed using the reference sequences, and 18s RNA was used for normalization (Supplementary Table S1). qRT-PCR was performed using a 20 μL reaction system with a procedure as follows: 95 °C for 30 s, followed by 39 cycles of 95 °C for 5 s, and 60 °C for 30 s. Melting curves were generated after each run to ensure a single PCR product. Each reaction was run in triplicate. The mRNA quantity of each gene was calculated with the 2−ΔΔCT method [31]. 

### 2.7. Statistical Analysis 

In this study, all experiments were executed in triplicate, and the obtained data represented the means ± S.E. The one-way analysis of variance (ANOVA) and Tukey’s multiple range tests were used to evaluate the difference between groups.

## 3. Results

### 3.1. Summary of Transcriptome Sequence and Assembly 

**Figure 1:**
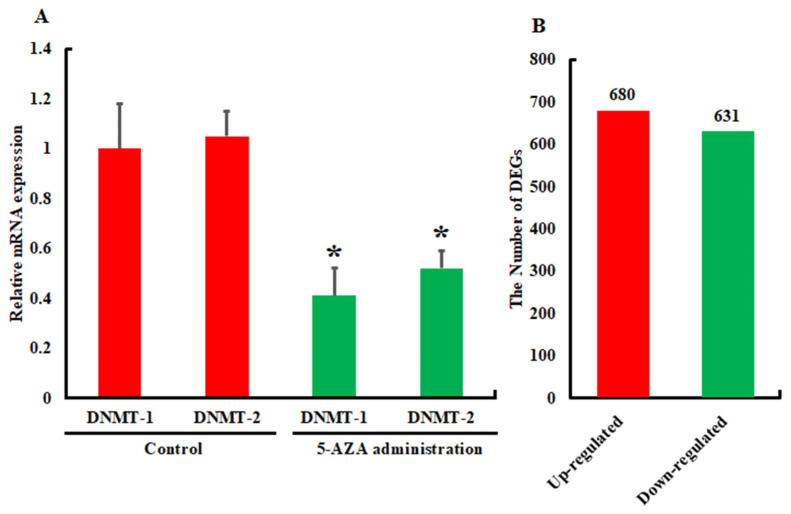
The inhibition of DNMTs and the comparison of the number of differentially expressed genes. (**A**): The inhibition of DNMTs analyzed by the qRT-PCR. (**B**): The levels of gene expression in 5-AZA- and bacterial-challenged groups compared to those in the control group. The differentially expressed genes were screened out by |log fold change (FC)| ≥ 1. Bars show the mean ± S.E. (n = 3), and asterisks indicate significant differences (* *p* < 0.05).

To investigate the effect of the DNA methylation inhibitor 5-AZA, two groups of pupae (5-AZA-treated and a control group) were sampled to collect fat body tissue after bacterial (*B. cereus*) treatment. We also examined the suppression of DNMTs prior to bacterial treatment (Figure 1A) and then sequenced the collected samples, with three replicates included in each group. After filtering the data, the transcriptome sequence generated 30,212,355, 24,056,953, and 25,478,892 clean reads from the 5-AZA-Gram-positive-bacteria (*B. cereus*)-infected group and 24,534,056, 26,678,456, and 23,536,596 from the control group (Table 1). The obtained sequenced data were assembled de novo using paired-end raw reads generated by the Illumina HiSeq2500 instrument. Quality evaluation was carried out in accordance with Illumina guidelines, and it was found that >95% and >92% of the sequencing data for each experimental group (treated with 5-AZA and bacteria) and control group (treated with only bacteria), respectively, had Q20 and Q30 quality scores. The contents for each experimental and control group were 43.95%, 43.00%, 42.21% and 40.95%, 42.52%, and 42.58%, respectively (Table 1). As shown in Table 2, 117,272 transcripts were generated using Trinity software with a mean length of 1425.323 bp and an N50 length of 2357 bp. A total of 55,131 unigenes were generated with an average length of 977.2239 bp and an N50 length of 1621 bp. Among these unigenes, 26,435 (47.95%) were in the range of 300−500 bp, 14,437 (26.19%) were 500−1000 bp, 7620 (13.82%) were 1−2 kbp, and 6638 (12.04%) were longer than 2 kbp. These observations propelled us to conclude that the obtained data are of high quality, and thus, the unigenes can be further used for annotation analysis. There were a total of 27,361 unigenes with 8187, 12,562, 13,351, 17,920, 19,325, 14,013, 25,402, and 23,492 annotated COG, GO, KEGG, Pfam, Swiss-Prot, eggNOG, and NCBI_nr, respectively (Supplementary Table S2).

### 3.2. Analysis of Differentially Expressed Genes 

**Figure 2:**
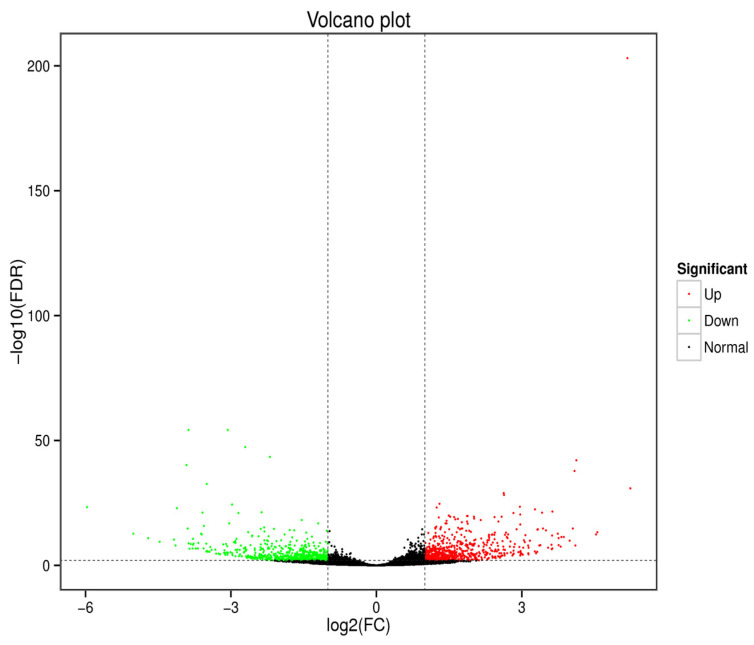
Volcano plot of the differentially expressed genes. The fold change of gene expression between the 5-AZA + bacterial treated group and the control group is represented by the horizontal ordinate. The vertical ordinate exhibits the statistical significance of the change in gene expression. Each gene is represented by a point on the plot, with the blue and red points representing the genes that were significantly down-regulated and up-regulated, respectively.

The expression patterns of genes in the 5AZA + bacteria (*B. cereus*)-injected group were compared with the control samples. In the comparison, it was found that by applying the standard threshold of *p* ≤ 0.001, false discovery rate ≤ 0.001, and |log fold change (FC)| ≥ 1, a total of 680 up-regulated and 631 down-regulated DEGs were screened out (Figure 1B and Figure 2).

**Figure 3:**
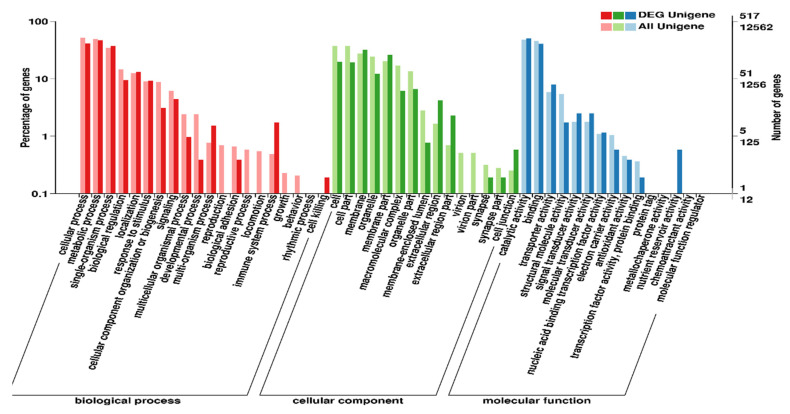
The top 25 GO terms in biological process cellular components, and molecular function categories with enriched in the differentially expressed gene. The Y-axis corresponds to the number of DEGs, whereas the X-axis shows different gene functions.

To understand the functional classification of DEGs, the unigene sequences were aligned against the GO and COG databases. The DEGs were classified into cellular components, molecular functions, and biological processes. The biological process groups possessed 20 subcategories, while cellular component and molecular function groups represented 15 subcategories each. Among the biological processes, cellular processes, metabolic processes, and the single-organism metabolic process, the genes were greatly expressed, whereas the cell, cell part, membrane, and organelle genes were highly expressed in cellular components. Finally, catalytic activity and binding genes were the most abundant among the molecular functions (Figure 3, Supplementary Table S3). 

**Figure 4:**
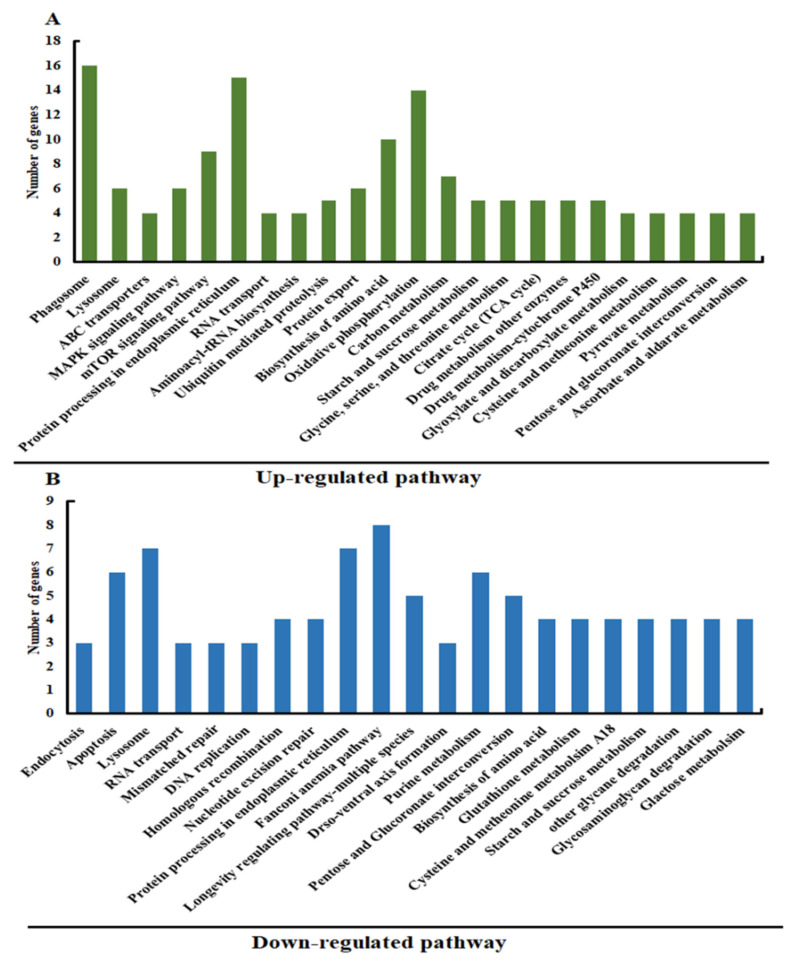
(**A**): The top 21 enriched pathways for the up-regulated genes. (**B**): The top 21 enriched pathways for the down-regulated genes.

The Eukaryotic Orthologous Groups (KOG) database categorizes homologous gene products, and each KOG protein is presumed to be derived from an ancestral protein. Based on the KOG functional classification, overall unigenes were mapped into 21 KOG categories, with general function prediction only. This was followed by signal-transduction mechanisms and posttranslational modifications, protein turnover, chaperones, and defense mechanisms. This suggests that unigenes may be involved in a variety of functions, especially immune defense in *A. pernyi* (Figure 1B). For the up-regulated unigenes, the most enriched pathways were phagosome, protein processing in the endoplasmic reticulum, oxidative phosphorylation, and the biosynthesis of amino acids (Figure 4A, Supplementary Table S4). The Fanconi anemia pathway, the lysosome, purine metabolism, and the apoptosis pathways were among the genes that were down-regulated (Figure 4B, Supplementary Table S4).

### 3.3. Validation and Reliability of the Transcriptome Data by qRT-PCR

**Figure 5:**
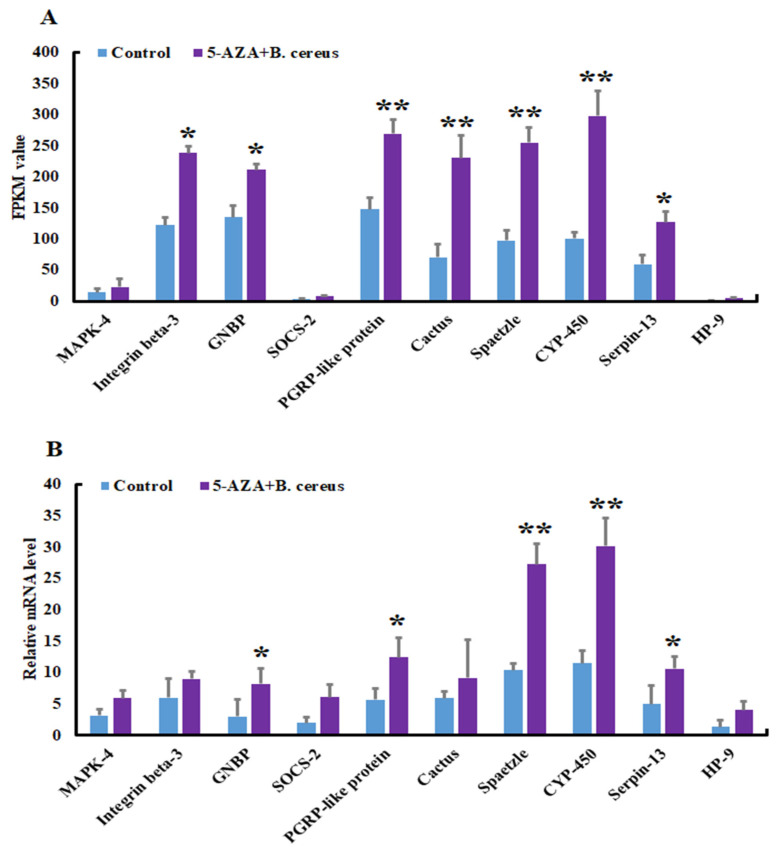
The expression of selected differentially expressed genes for the validation of data. (**A**): Expression of 10 selected unigenes expression (FPKM value) in the experimental and control group. (**B**): Quantitative RT-PCR verification of RNA sequence data. X-axis and Y-axis show the 10 selected differentially expressed genes and the relative expression (*B. cereus*-injected vs. 5-AZA+*B. cereus*-injected), respectively. The sequences include c89459.graph_c0, c92066.graph_c0, c90624.graph_c0, c91406.graph_c2, c93759.graph_c1, c93774.graph_c0, c77163.graph_c1, c85165.graph_c0, c89772.graph_c1, c85340.graph_c0 encoding mitogen-activated protein kinase kinase 4, integrin beta pat-3-like, GNBP, suppressor of cytokine signaling 2-like isoform X2, peptidoglycan recognition protein-like protein, cactus, spaetzle, cytochrome P450, serine protease inhibitor 13 precursor, and hemolymph proteinase 9. (* *p* < 0.05, ** *p* < 0.01).

First, we searched for the housekeeping gene actin in both the experimental (5-AZA+*B. cereus*) and control groups to ensure that the data were reliable before proceeding. We found that the expression of this housekeeping gene was the same in both groups. After confirming housekeeping gene expression, we selected 10 DEGs from *A. pernyi* related to the innate immune system and with different signaling pathways. In order to validate the expression profiles in both the experimental and control groups, we performed qRT-PCR analysis. The results revealed that the expression profiles of the candidate genes were consistent with those of the RNA-seq data (Figure 5), indicating that the RNA sequencing data were reliable. These findings will be useful in improving our understanding of the process of DNA methylation and its effect on gene expression.

## 4. Discussion

Unlike mammals, where DNA methylation has extensively been reported, many insect species have been shown to be devoid of the DNA-methylation process [22]. When it comes to most insect species, there is currently a dearth of literature relating to the existence of this epigenetic phenomenon. Therefore, our understanding of DNA methylation and its involvement in various physiological responses is extremely limited at this time. In mammals, the biological roles of DNA methylation have been extensively investigated; however, in insects, this information is still in its early stages. To investigate the biological roles of DNA methylation in *A. pernyi* innate immunity, the differential expression of genes related to immunity was evaluated after applying 5-AZA and bacteria. The suppression of DNMTs was confirmed by qRT-PCR. Interestingly, in addition to DNMT-1, DNMT-2 expression was also significantly reduced. Previous studies on *Drosophila melanogaster, Dictyostelium discoideum*, and *Entamoeba histolytica* showed that DNMT-2 could regulate DNA methylation [32,33,34] in addition to tRNA methylation [35]. There are multiple lines of evidence suggesting that 5-AZA, as a ribonucleoside, can also form covalent bonds with human and mouse DNMT-2 homologs and has the ability to inhibit its expression [35,36,37]. For this purpose, transcriptome sequencing was used to compare the genes in the DNA-methylation-inhibitor-bacteria infected with the control. In this study, we focused on the immune-related DEGs and reported that the inhibition of DNA methylation has an effect on the expression of genes in insects. 

Insects have a complex immune system that is divided into humoral and cellular immunity components. Cellular immunity protects insects from pathogen infection through encapsulation, phagocytosis, and nodulation, while humoral immune responses include the production of antimicrobial peptides [38,39]. In the present study, from the RNA sequence data, we observed that several DEGs encoding immune-related proteins regulating cellular and humoral immunity were up-regulated (680 unigenes) with variable expression. Among these up-regulated, we selected 10 DEGs (c89459.graph_c0, c92066.graph_c0, c90624.graph_c0, c91406.graph_c2, c93759.graph_c1, c93774.graph_c0, c77163.graph_c1, c85165.graph_c0, c89772.graph_c1, c89772.graph_c1, c85340.graph_c0 encoding mitogen-activated protein kinase kinase kinase 4, integrin beta pat-3-like, GNBP, suppressor of cytokine signaling 2-like isoform X2, peptidoglycan recognition protein-like protein, cactus, spaetzle, cytochrome P450, serine protease inhibitor 13 precursor, hemolymph proteinase 9) representing different signaling pathways to validate the RNA sequence data and to highlight the importance of DNA methylation in the physiological processes of insects. A growing body of evidence suggests that DNA methylation, in particular cytosine (CpG) methylation, is a critical and reversible gene-regulation mechanism. DNA methylation controls transcriptional modulation by influencing the recruitment of regulatory factors to gene promoters [40]. Many studies have reported that microbial infection induces genome DNA methylation and that this is linked with host immune responses [41,42]. For example, Laurson [43] reported that human papillomavirus infection induces aberrant host DNA methylation through direct interaction of the viral protein E7 with the DNA methylation enzyme DNMT-1 [43]. *Escherichia coli* infection regulates the transcription patterns of DNMT-1, and inducing CpG hypermethylation reduces the expression of the cell-cycle inhibitor CDKN2A in human uroepithelial cells [44]. Similarly, *Mycobacterium tuberculosis* infection causes fast methylation of host DNA at distal enhancer elements and associated chromatin remodeling [45]. Kausar et al. [22] recently reviewed that, despite the fact that DNA methylation levels in insects are low, DNA methylation has the ability to modify a variety of physiological processes, including the innate immune system. From these epigenetic effects of microbial infections and subsequent modification of the innate immune response, it is clear that DNA methylation plays a role in the interactions between host and pathogen.

Based on our RNA sequencing data, we found that inhibiting DNA methylation could have an impact on the insect host–pathogen interaction. The Toll (GNBP, cactus, serine protease inhibitor protein, hemolymph protease 9, Spatzle), IMD (peptidoglycan recognition protein), and JAK-STAT (suppressor of cytokine signaling 2) signaling pathways are all important in the innate immunity of insects. In response to Gram-negative bacterial infections and viruses, IMD and JAK-STAT are activated, whereas the Toll pathway is activated in response to Gram-positive bacteria and fungi [46,47,48]. Following bacterial invasion, pattern-recognition receptors are sensed by GNBP, which then activates Spatzle, which plays a vital role in the activation of the Toll pathway. The activated Spatzle binds to Toll receptors for the activation of the downstream cascade [48]. Cactus is phosphorylated and cleaved off Dif late in this process, allowing Dif to translocate into the nucleus and modulate the transcription of effector genes [49]. Serine protease inhibitors and hemolymph protease control the melanization process of the hemolymph to attenuate pathogens [39]. The peptidoglycan-recognition protein has a strong affinity for Gram-positive bacteria and can also bind with Gram-negative bacteria to initiate signaling via IMD and the Toll pathway [49], whereas the suppressor of cytokine 2 is a well-known negative regulator of the JAK-STAT pathway, which checks the activity of this pathway during infection and development [50,51,52,53]. These pathways mainly attenuate microbial infection via inducing the production of antimicrobial peptides [39,49]. In the present study, when *A. pernyi* was exposed to a DNA methylation inhibitor and a Gram-positive bacterial challenge, we observed that genes associated with the Toll, IMD, and JAK-STAT pathways were increased in comparison to the control group. This suggests that DNA methylation inhibition induced robust immune responses in *A. pernyi*. 5-AZA, a commonly used DNA methylation inhibitor, seems to impair the production of DNA-methylation-associated genes, particularly DNMTs. This impairment inhibits the methylation status at the gene loci, which may affect the expression of genes [23]. 

Evidence suggests that these three pathways are interlinked and that they are activated in order to reduce microbial infection. For example, according to Li and Xiang [54], the Toll pathway in invertebrates is activated not only by Gram-positive bacteria but also by Gram-negative bacteria and the WSSV virus. In addition, Hedengren-Olcott et al. [55] suggested that the activation pathways seem to be specific to bacterial strains. They went on to say that some bacteria can induce both the Toll and IMD pathways, suggesting that there is some crosstalk between these immune-signaling pathways. Additionally, integrin, a cell surface receptor protein that has been reported to contribute to encapsulation, pathogen adherence, and phagocytosis, was increased in addition to these immune-pathway-related genes [56,57]. Here, we discussed our results very precisely because our data showed that the inhibition of DNA methylation has an impact on a variety of physiological processes, such as development. Altogether, these results suggest that pathogen infection may induce DNA methylation of the host genome, which may suppress immune responses by yet-unknown mechanisms. On the other hand, our data suggest that inhibiting DNA methylation could induce a robust immune response in insects, thereby shaping the host–pathogen interaction. 

## 5. Conclusions

In conclusion, our findings imply that DNA methylation probably occurs in the Chinese oak silkworm, *A. pernyi*, similar to that of *B. mori*. We also identified a DNA methylation tool kit in this species (data not shown), which further ensured that the *A. pernyi* genome is methylated. In addition, in this species, inhibition of DNA methylation might result in the induction of the expression of genes, especially immune-related genes, which can influence the host–pathogen interaction. This study showed that DNA methylation inhibition caused modifications in a number of signaling pathways. Thus, this study provides a repository to investigate the level of DNA methylation in the genome of the insect, and subsequently, which genes promoted are methylated and how DNA methylation modifies different physiological processes, including development and immunity. 

## Figures and Tables

**Table 1:** Statistical analysis of the transcriptome sequence data.

	Clean Bases	Clean Reads	Mapped Reads	Mapped Ratio	Q20%	Q30%	GC (%)
5-AZA and bacteria injected	8,970,065,914	30,212,355	23,677,535	78.37	97.80	93.81	43.95
5-AZA and bacteria injected	7,182,585,608	24,056,953	19,384,242	80.58	97.53	93.41	43.00
5-AZA and bacteria injected	7,613,303,876	25,478,892	20,665,639	81.11	97.50	93.34	42.21
Only bacteria injected	7,333,560,222	24,534,056	19,994,004	81.49	97.48	93.20	40.95
Only bacteria injected	7,653,093,914	26,678,456	20,316,290	79.12	97.98	94.21	42.52
Only bacteria injected	7,018,186,154	23,536,596	18,881,662	80.22	97.73	93.69	42.58

**Table 2:** Distribution of splicing length.

Length Range	Transcript	Unigene
300–500	35,064 (29.90%)	26,435 (47.95%)
500–1000	28,596 (24.38%)	14,437 (26.19%)
1000–2000	25,629 (21.85%)	7620 (13.82%)
2000+	27,981 (23.86%)	6638 (12.04%)
Total Number	117,272	55,131
Total Length	167,150,431	53,875,332
N50 Length	2357	1621
Mean Length	1425.323	977.2239

## Data Availability

The data presented in this study are available in article and Supplementary Material.

## Supplementary Materials

The following supporting information can be downloaded at: https://www.mdpi.com/article/10.3390/insects13030296/s1, Table S1: Gene specific Primers used for qRT-PCR analysis.; Table S2: Summary Statistics of the *A. pernyi* Transcriptome Annotation.; Table S3: Table of GO Enrichment of All Genes and DEGs.; Table S4: Classification of the Unigenes Annotated in KOG.
