# Supplementary material for: Transcriptome Sequencing Highlights the Regulatory Role of DNA Methylation in Immune-Related Genes’ Expression of Chinese Oak Silkworm, Antheraea pernyi
Source: Insects. 2022 Mar 17;13(3):296. doi: 10.3390/insects13030296 (PMC8951095; doi:10.3390/insects13030296)
Supplement: Supplementary file 1 [file insects-13-00296-s001.zip › insects-1581905-supplementary.pdf]

Table S1. Gene specific Primers used for qRT-PCR analysis.

Gene ID	Gene name	Forward/reverse	Primer sequence (5′-3′)
c89459.graph_c0	Mitogen-activated protein kinase kinase kinase 4	F	GATGATCCGGTCGAAGTGTT
		R	CGGAAGGAGAGGTAGTCGTG
c92066.graph_c0	Integrin beta pat-3-like	F	GTCCAGGACAATTGCAGGAT
		R	CGTGGCTGTCTTGTCTTTCA
c90624.graph_c0	GNBP	F	TCGGTCGGCTCAGAGACTAT
		R	GATACGTGGTGACTGCAGGA
c91406.graph_c2	Suppressor of cytokine signaling 2-like isoform X2	F	AAAGGAGTCGGGTGTGTTTG
		R	CGGCAGCTTTTTGTAACGAT
c93759.graph_c1	Peptidoglycan recognition protein-like protein	F	CGCCATCTTGGTTACGTTTT
		R	CGGCAGCCATTTTGTATCA
c93774.graph_c0	Cactus	F	GACGTGAAGCTGGTGCAGTA
		R	AGAGGCTTTCGCTTTCACTG
c77163.graph_c1	Spaetzle	F	TGTACCGTGAAACCACCAGA
		R	GGCGGACCTTTTGAATGTAA
c85165.graph_c0	Cytochrome P450	F	GTTGGCCGTCGACAATAAAT
		R	GGCTTGTATTCGGGAGATCA
c89772.graph_c1	Serine protease inhibitor 13 precursor	F	CGCTAAAAGCACACGCTACA
		R	GGCTTCTGGTAACGTGCATT
c85340.graph_c0	Hemolymph proteinase 9	F	ATCTGGTTCGTCGATTACGG
		R	TATAGAGGCGCTGGAGCAGT
	Ap18s rRNA	F	CGATCCGCCGACGTTACTACA
		R	GTCCGGGCCTGGTGAGATTT
	DNMT1	F	ACGACTACGCCGTTGGTAAG
		R	TCATCAGCTTGTTCCACAGC
	DNMT2	F	TGTGCATGGAATGAATCAGG
		R	CGGATCATTTTCATCGAGGT

Table S2. Summary Statistics of the *A. pernyi* Transcriptome Annotation.

Annotated databases	Annotated number	Percentage %	300 ≤ length < 1000	length ≥ 1000
COG_Annotation	8187	29.92	4222	3965
GO_Annotation	12562	45.91	6934	5628
KEGG_Annotation	13351	48.79	7523	5828
KOG_Annotation	17920	65.49	10046	7874
Pfam_Annotation	19325	70.62	10409	8916
Swissprot_Annotation	14013	51.21	7045	6968
eggNOG_Annotation	25402	92.84	15144	10258
nr_Annotation	23492	85.85	12893	10599
All_Annotated	27361	100	16565	10796

Table S3. Table of GO Enrichment of All Genes and DEGs.

Term Type	Subcategories	All Genes	DEGs
**Biological Process**			
	Cellular process	6469	126
	Metabolic process	6261	137
	Single-organism process	4371	122
	Biological regulation	1828	33
	Localization	1566	53

	Response to stimulus	1142	30
	Cellular component organization or biogenesis	1099	8
	Signaling	770	13
	Multicellular organismal process	307	4
	Developmental process	304	2
	Multi-organism process	96	8
	Reproduction	88	0
	Biological adhesion	84	1
	Reproductive process	74	0
	Locomotion	69	0
	Immune system process	62	9
	Growth	29	0
	Behavior	26	0
	Rhythmic process	3	0
	Cell killing	1	1
**Cellular Component**			
	Cell	4692	67
	Cell part	4634	65
	Membrane	3476	118
	Organelle	3079	38
	Membrane part	2571	101
	Macromolecular complex	2118	22
	Organelle part	1699	24
	Membrane-enclosed lumen	354	4
	Extracellular region	206	17
	Extracellular region part	87	8
	Virion	64	0
	Virion part	64	0
	Synapse	40	0
	Synapse part	35	0
	Cell junction	32	1
**Molecular Function**			
	Catalytic activity	6031	155
	Binding	5788	120
	Transporter activity	730	31
	Structural molecule activity	676	5
	Molecular transducer activity	225	7
	Signal transducer activity	225	7
	Nucleic acid binding transcription factor activity	137	5
	Electron carrier activity	132	3
	Antioxidant activity	57	1
	Transcription factor activity, protein binding	46	1
	Protein tag	11	0
	Metallochaperone activity	4	0
	Nutrient reservoir activity	3	2
	Chemoattractant activity	2	0
	Molecular function regulator	1	0

Table S4. Classification of the Unigenes Annotated in KOG.

Class Name	Abbreviations	Numbers	Percentage
Translation, ribosomal structure and biogenesis	J	13	6.10
RNA processing and modification	A	1	0.47
Transcription	K	2	0.94
Replication, recombination and repair	L	3	1.41

Chromatin structure and dynamics	B	0	0
Cell cycle control, cell division, chromosome partitioning	D	0	0
Nuclear structure	Y	0	0
Defense mechanisms	V	10	4.70
Signal transduction mechanisms	T	13	6.10
Cell wall/membrane/envelope biogenesis	M	8	3.76
Cell motility	N	0	0
Cytoskeleton	Z	0	0
Extracellular structures	W	0	0
Intracellular trafficking, secretion, and vesicular transport	U	3	1.41
Posttranslational modification, protein turnover, chaperones	O	29	13.6
Energy production and conversion	C	14	6.57
Carbohydrate transport and metabolism	G	26	12.21
Amino acid transport and metabolism	E	11	5.16
Nucleotide transport and metabolism	F	5	2.35
Coenzyme transport and metabolism	H	7	3.29
Lipid transport and metabolism	I	16	7.51
Inorganic ion transport and metabolism	P	9	4.23
Secondary metabolites biosynthesis, transport and catabolism	Q	13	6.10
General function prediction only	R	23	10.80
Function unknown	S	3	1.41
Mobilome: prophages, transposons	X	4	1.88

Footnote: Functional classification of unigenes are classified from A-Z
